# Marjoram Relaxes Rat Thoracic Aorta Via a PI3-K/eNOS/cGMP Pathway

**DOI:** 10.3390/biom9060227

**Published:** 2019-06-11

**Authors:** Adnan Badran, Elias Baydoun, Ali Samaha, Gianfranco Pintus, Joelle Mesmar, Rabah Iratni, Khodr Issa, Ali H. Eid

**Affiliations:** 1 Department of Nutrition, University of Petra, Amman, P.O. Box 961343, Amman 11196, Jordan; abadran@uop.edu.jo; 2 Department of Biology, American University of Beirut, Beirut P.O. Box 11-0236, Lebanon; eliasbay@aub.edu.lb (E.B.); joellemesmar@gmail.com (J.M.); 3 Department of Biomedical Sciences, Lebanese International University, Beirut, P.O. Box 146404 Mazraa, Lebanon; ali.samaha@liu.edu.lb; 4 Faculty of Public Health IV, Lebanese University, Beirut, P.O. Box 6573/14 Badaro, Lebanon; 5 Department of Biomedical Sciences, Qatar University, Doha P.O. Box 2713, Qatar; gpintus@qu.edu.qa; 6 Biomedical Research Center, Qatar University, Doha P.O. Box 2713, Qatar; 7 Department of Biology, United Arab Emirates University, Al Ain P.O. Box 15551, UAE; r_iratni@uaeu.ac.ae; 8 Department of Pharmacology and Toxicology, American University of Beirut, Beirut P.O. Box 11-0236, Lebanon

**Keywords:** marjoram, hypertension, vasorelaxation, PI3-K, nitric oxide, cGMP

## Abstract

Despite pharmacotherapeutic advances, cardiovascular disease (CVD) remains the primary cause of global mortality. Alternative approaches, such as herbal medicine, continue to be sought to reduce this burden. *Origanum majorana* is recognized for many medicinal values, yet its vasculoprotective effects remain poorly investigated. Here, we subjected rat thoracic aortae to increasing doses of an ethanolic extract of *Origanum majorana* (OME). OME induced relaxation in a dose-dependent manner in endothelium-intact rings. This relaxation was significantly blunted in denuded rings. N(ω)-nitro-l-arginine methyl ester (L-NAME) or 1H-[1,2,4]oxadiazolo[4,3,-a]quinoxalin-1-one (ODQ) significantly reduced the OME-induced vasorelaxation. Cyclic guanosine monophosphate (cGMP) levels were also increased by OME. Moreover, wortmannin or LY294002 significantly reduced OME-induced vasorelaxation. Blockers of ATP-sensitive or Ca^2+^-activated potassium channels such as glibenclamide or tetraethylamonium (TEA), respectively, did not significantly affect OME-induced relaxation. Similarly, verapamil, a Ca^2+^ channel blocker, indomethacin, a non-selective cyclooxygenase inhibitor, and pyrilamine, a H1 histamine receptor blocker, did not significantly modulate the observed relaxation. Taken together, our results show that OME induces vasorelaxation via an endothelium-dependent mechanism involving the phosphoinositide 3-kinase (PI3-K)/ endothelial nitric oxide (NO) synthase (eNOS)/cGMP pathway. Our findings further support the medicinal value of marjoram and provide a basis for its beneficial intake. Although consuming marjoram may have an antihypertensive effect, further studies are needed to better determine its effects in different vascular beds.

## 1. Introduction

Cardiovascular disease (CVD) continues to cause more mortalities than any other disease [1]. Despite major pharmacotherapeutic advances, chronically elevated blood pressure persists as a major contributor to CVD-associated mortality [2]. Although many antihypertensive drugs continue to be developed, these synthetic drugs are not without many deleterious adverse effects. In addition to these synthetic drugs, the use of herbal medicine and plant-based therapies is increasing in many countries [3]. In recent years, more interest has been given to these therapies, largely due to their ease of availability, presumed low toxicity, and documented benefits [4,5,6,7,8]. Indeed, many of these plants have been employed for the particular treatment or management of cardiovascular disease [5,6,7,8,9].

*Origanum majorana* L. is a perennial subshrub with a distinguished fragrant foliage. It is native to southern Europe and the Mediterranean. It has been traditionally used as a folk medicine for many ailments, such as indigestion, headache and asthma [10,11]. Interestingly, the genus *Origanum* possesses bountiful biological properties, such as antioxidant, anti-inflammatory and anticholinesterase effects, as well as protection against aging and neurodegenerative disease [12]. In addition, marjoram elicits an in vitro microbicidal activity [13,14]. We have also shown that marjoram inhibits the malignant phenotype of breast cancer cells [15,16]. Relevantly, it has been reported that marjoram is used in the management/treatment of hypertension, although the mechanism of action was not elucidated [17].

Bioactives of marjoram, such as the monoterpene carvacrol, exhibit a potent inhibitory effect against bacterial and fungal infections, as well as antispasmodic effects [13]. Moreover, carvacrol can quench free radicals and ameliorate hypercontraction of aortic vasculature [18]. More importantly, carvacrol is known to play an important role in endothelium-dependent vasorelaxation of rat aortae via a mechanism that apparently involves potassium and calcium channels [19,20]. Similarly, thymol, another monoterpe found in marjoram, appears to induce relaxation of isolated rat aortae [20]. Hesperetin, another bioactive of marjoram [21], reduces systolic blood pressure in spontaneously hypertensive rats via an endothelium-dependent mechanism [22].

Despite these beneficial values of *O. majorana* or its bioactives, and to the best of our knowledge, no studies have so far investigated the vascular activity caused by *O. majorana*. Accordingly, the present study was undertaken to investigate and evaluate the potential vasorelaxatory effects of *O. majorana* on isolated rat aortae.

## 2. Materials and Methods

### 2.1. Preparation of the Extract

*Origanum majorana*, locally grown and commonly available, was collected in August 2014. Dried leaves were ground with a mortar and pestle, and the powder extracted with 70% EtOH for 3 h in a reflux apparatus at 70 ± 5 °C. Following filtration, the extract was collected and evaporated at 60 °C, and then it was lyophilized in a freeze-dryer to obtain a powder of the crude extract. This powder was weighed and kept at −20 °C until further use.

### 2.2. Animals

The study protocol was approved by the scientific committee on animal care and use in the Faculty of Public Health at the Lebanese University (Permit number for Samaha Marjoram Project: UL/FSPIV/07/2011). Experiments were performed in conformity with the National institutes of health legislation on the use of laboratory animals. Every effort was taken to minimize animal suffering. Male Sprague-Dawley (SD) rats (230–290 g body weight; 6–8 weeks old) were housed in an animal laboratory at an ambient temperature of 23 ± 2 ° C, under a 12 h:12 h dark: light cycle. Animals had access to a standard animal chow and tap water ad libitum.

### 2.3. Drugs and Chemicals

Acetylcholine, norepinephrine, tetraethylammonium (TEA), 1H-[1,2,4]oxadiazolo[4,3-alpha]quinoxalin-1-one (ODQ), atropine, 3-isobutyl-1-methylxanthine (IBMX), indomethacin, pyrilamine, verapamil, and Nω-nitro-l-arginine methyl ester (L-NAME) were purchased from Sigma-Aldrich Co. (St. Louis, MO, USA). LY294002 and Wortmannin was obtained from Tocris (Abingdon, UK) and Alexis Biochemicals (Lausen, Switzerland), respectively.

### 2.4. Arterial Ring Preparation and Vascular Reactivity

Male SD rats were euthanized by an overdose of pentobarbital (50 mg/kg body weight). Thoracic aortae were quickly dissected into a cold modified Krebs-Henseleit (KHB) buffer, as in our recent papers [7,8]. Aortae were cleaned of fat and other adhering connective tissues and cut into ring segments, which were suspended for the measurement of isometric force in organ chambers filled with KHB (pH 7.4), maintained at 37 °C, and bubbled with a gas mixture of 95% O_2_—5% CO_2_. The KH solution contained (in 10^−3^ M): NaCl (130), KCl (4.7), CaCl_2_*·*2H_2_O (1.6), Mg_2_SO_4_ 7H_2_O (1.17), KH_2_PO_4_ (1.18), NaHCO_3_ (14.9), and glucose (5.5).

During the equilibration period (at least 30 min), the rings underwent isometric resting tension in a stepwise manner, as we recently described [7,8]. Cumulative concentrations (0.01–1 mg/mL,) of *O. majorana* (OME) were added, where at each dose of OME, the response curve would reach a plateau before the subsequent dose was added. To determine the endothelium-dependent relaxation, aortic rings with denuded endothelium were pre-contracted with norepinephrine (NE; 3 µM) and then exposed to incrementally increased concentrations of OME. As we recently published [8], denudation was done by gently rubbing, in a to and from motion, the lumen of the ring with a roughened piece of stainless wire. Generation of less than 15% relaxation with acetylcholine is considered indicative of a successful denudation.

When assessing the potential role of nitric oxide (NO), endothelium-intact rings were pre-incubated with L-NAME (100 μM), a NO synthase inhibitor, for 30 min. Rings were then contracted with NE, after which OME was added.

When determining the role of prostaglandins, histamine H1-receptors, muscarinic receptors, guanylate cyclase, or potassium channels in OME-induced relaxation, the following inhibitors were used: indomethacin (10 µM), a non-selective cyclooxygenase inhibitor; pyrilamine (10 μM), a histamine H1-receptor antagonist or atropine (10 μM), a muscarinic receptor antagonist; oxadiazole quinoxaline (ODQ, 10 µM), a soluble guanylate cyclase inhibitor; glibenclamide (10 µM), an ATP-sensitive potassium channels blocker; or tetraethylammonium (100 µM), a non-selective inhibitor of Ca^2+^-activated potassium channels. When the role of calcium channels was to be assessed, and as previously described [7,8,23], rings were pre-incubated with verapamil hydrochloride (1µM), a blocker of L-type calcium channels before the addition of OME. 

To assess the potential role of phosphoinositide 3-kinase (PI3K)/Akt signaling, rings were pre-incubated with the PI3K inhibitors, LY294002 (10 µM) or wortmannin (100 nM), for 30 min. A cumulative dose response curve with increasing concentrations of OME followed.

To ensure that OME did not impart a significant irreversible effect on vascular responsiveness, aortic rings that had been exposed to OME were washed and exposed to norepinephrine (NE, 3 μM) or acetylcholine (ACh, 10 μM) anew. Finally, KCl (80 mM) was used to confirm viability of the rings.

### 2.5. cGMP Assay

Rings that had been pre-equilibrated were incubated in IBMX (a phosphodiesterase inhibitor; 0.1 mM) for 30 min before subsequent addition of NE. Then, these rings were re-equilibrated for an additional 30 min before adding OME. Tissues were then frozen in liquid nitrogen to stop the reaction, homogenized in trichloroacetic acid, and centrifuged at 10,000× *g* for 10 min. The supernatant was then extracted with water-saturated diethylether. Quantitation of protein or cyclic guanosine monophosphate (cGMP) in the extract was done by Bradford’s method or a specific immunoassay (GE Healthcare Life Sciences), Pittsburgh, PA, USA), respectively. Results are plotted as picomoles of cGMP per milligram of protein.

### 2.6. Graphing and Statistical Analysis

Graphing and determination of 50% effective dose (pED50) and *E*_MAX_, along with the confidence intervals, were performed by GraphPad Prism 6 software (GraphPad Software, San Diego, CA, USA). For statistical analysis, the same software package was used. Data are presented as means ± standard error (SE). Student’s *t*-test was used. If comparison between more than two means was needed, Analysis of variance (ANOVA) was used—either a one-way ANOVA with Tukey’s post hoc test or a two-way ANOVA with Sidak’s multiple comparison post hoc test. A *p*-value of less than 0.05 was considered statistically significant.

## 3. Results

### 3.1. Effect of Origanum majorana Extract on Norepinephrine-Induced Contraction 

**Figure 1:**
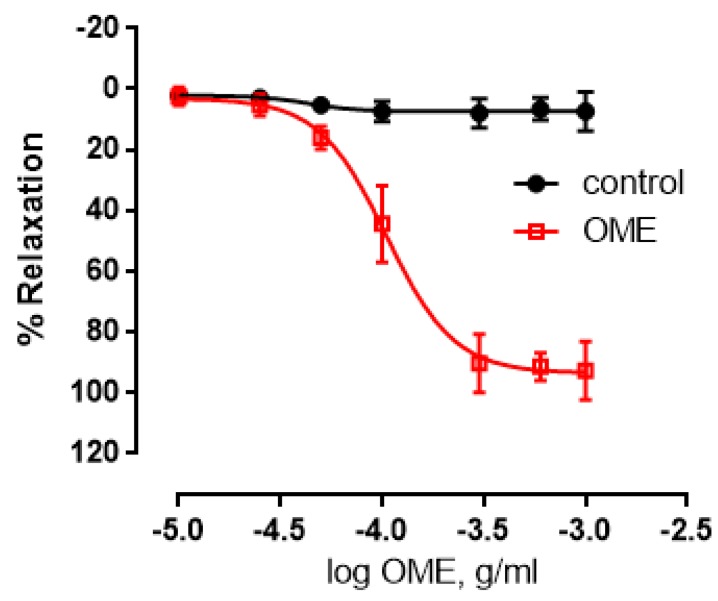
Effect of *Origanum majorana* (OME) extract on the vasorelaxation of aortic rings. Cumulative dose response curve for OME-induced relaxation of rat aortic rings was determined. Data expressed are mean ± standard error of the mean (SEM, *n* = 7).

We first determined the concentration-dependent vasorelaxatory effects of OME (0.001–1 mg/mL) on endothelium-intact aortic rings. OME caused a dose-dependent relaxation with the maximal relaxant effect being 90 ± 5%, and a pED_50_ of −3.98, with a 95% confidence interval of −4.0 to −3.9 (Figure 1). The vehicle (ethanol) did not impart any significant effect on the rings.

### 3.2. Effect of Origanum majorana Extract on Endothelium-Intact or Endothelium-Denuded Aortic Rings Pre-Contracted with Norepinephrine

**Figure 2:**
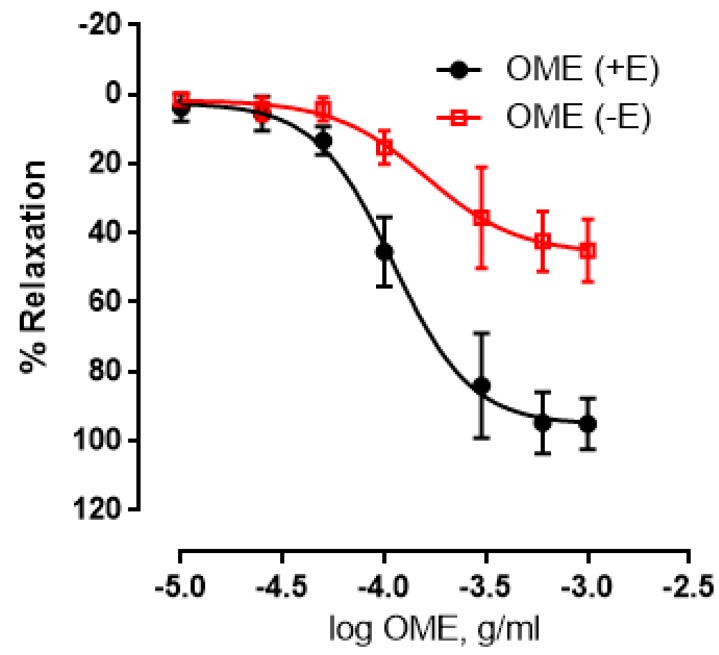
Role of endothelium in OME-induced relaxation. Cumulative dose-response curves for OME in isolated norepinephrine (NE)-pre-contracted rat aortic rings either with intact (+E; black) or denuded endothelium (-E; red). Data are expressed as mean ± SEM (*n* = 7; *p* < 0.01 for +E vs. -E).

We next wished to determine the potential role of endothelium in OME-induced relaxation. In both endothelium-intact and endothelium-denuded aortic rings, OME caused a dose-dependent relaxation (Figure 2). The maximal relaxant effect was 95 ± 3% or 46 ± 4.0% for endothelium-intact or endothelium-denuded aortic rings, respectively (Figure 2). A pED_50_ of −3.96 with a 95% confidence interval of −4.0 to −3.9 was obtained for the endothelium intact rings, while a pED_50_ of −3.79 with a 95% confidence interval of −3.9 to −3.6 was obtained for the endothelium-denuded ones. This significant difference between the two conditions clearly demonstrates that endothelium is, at least in part, involved in OME-induced relaxation.

### 3.3. Role of Nitric Oxide and cGMP in Origanum majorana Extract-Induced Aortic Relaxation

**Figure 3:**
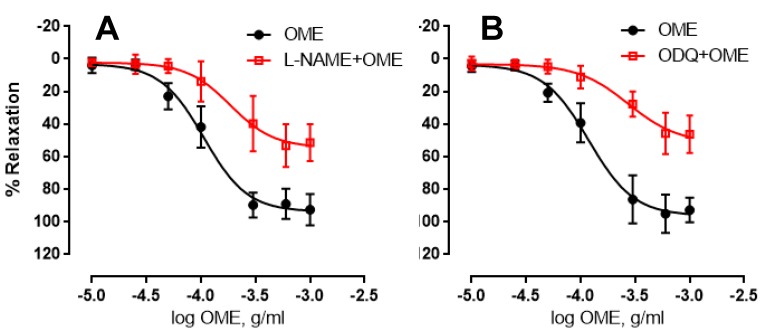
Role of nitrous oxide (NO) or cGMP in OME-induced relaxation. (**A**) Endothelium-intact rings were treated with cumulative doses of OME in the presence (red) or the absence (black) of Nω-nitro-l-arginine methyl ester (L-NAME) (inhibitor of eNOS, 100 µM). Data represent mean ± SEM (*p* < 0.01 for OME alone vs. L-NAME plus OME; *n* = 7). (**B**) Endothelium-intact rings were treated with cumulative doses of OME in the presence (red) or absence (black) of ODQ (inhibitor of soluble guanylate cyclase, 1 µM). Data represent mean ± SEM (*p* < 0.01 for OME vs. ODQ plus OME; *n* = 6).

NO and cGMP are two important signalling molecules that play a vital role in vasorelaxation. We next investigated the role of these molecules in the vasorelaxant effect of OME. OME-induced relaxation of endothelium-intact aortic rings was significantly diminished by the presence of L-NAME or ODQ. In the presence or absence of L-NAME, the maximal relaxant effect was 54 ± 5% or 94 ± 3%, respectively (Figure 3A), and pED_50_ was −3.7 or −3.9 with confidence intervals of −3.9 to −3.5 or −4 to −3.9, respectively. Likewise, the maximal relaxant effect in the presence or absence of ODQ was 52 ± 7% or 96 ± 4%, respectively (Figure 3B), and pED_50_ was −3.5 or −3.9 with confidence intervals of −3.7 to −3.3 or −4 to −3.8, respectively. Taken together, these data support the notion that both NO and cGMP play a significant role in OME-induced vasorelaxation. 

### 3.4. Effect of Origanum majorana Extract on the Production of cGMP in Aortic Rings 

**Figure 4:**
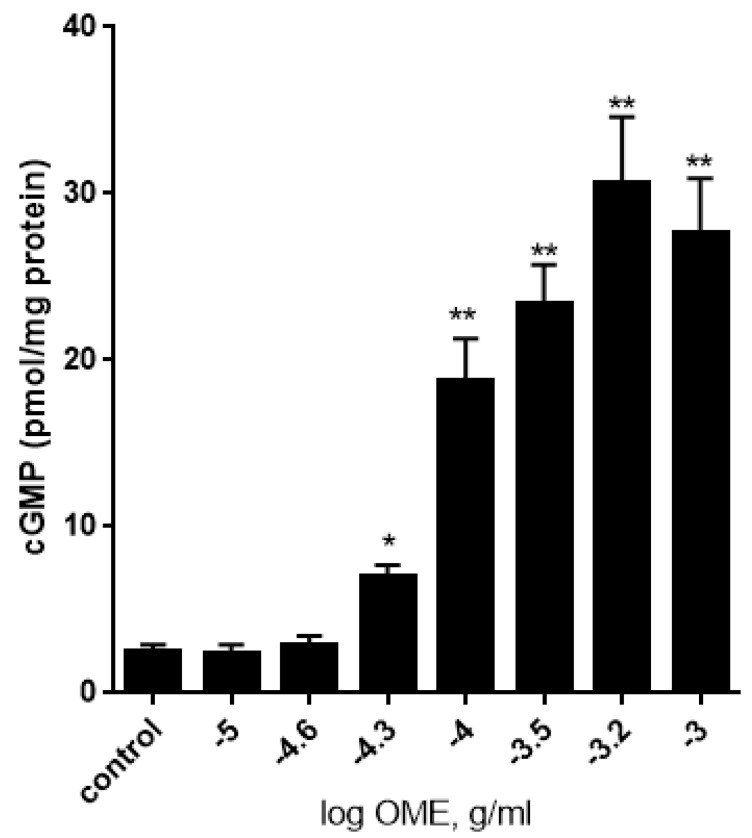
Effect of OME on cGMP levels. Rings were treated without (control) or with increasing concentrations of OME. cGMP levels were quantitated by an immunoassay. Data are shown as mean ± SEM, *n* = 5. (* *p* < 0.01; ** *p* < 0.001).

Because OME-induced vasorelaxation was significantly reduced by L-NAME and ODQ, we next wished to determine if OME modulates the level of cGMP. Treatment with OME caused a significant and dose-dependent increase in the levels of cGMP (Figure 4). In control rings (vehicle-treated), the cGMP level was 2.8 ± 0.8 (mean ± standard error of the mean (SEM)) picomole/mg protein. However, in rings treated with 0.3 mg/mL OME, the cGMP level rose to a dramatic level of 28 ± 5 (mean ± SEM) picomole/mg protein (*p* < 0.001). Importantly, L-NAME and ODQ significantly inhibited OME-induced production of cGMP (data not shown). 

### 3.5. Involvement of the PI3K Signaling Pathway in the Origanum majorana Extract-Induced Relaxation of Aortic Rings 

**Figure 5:**
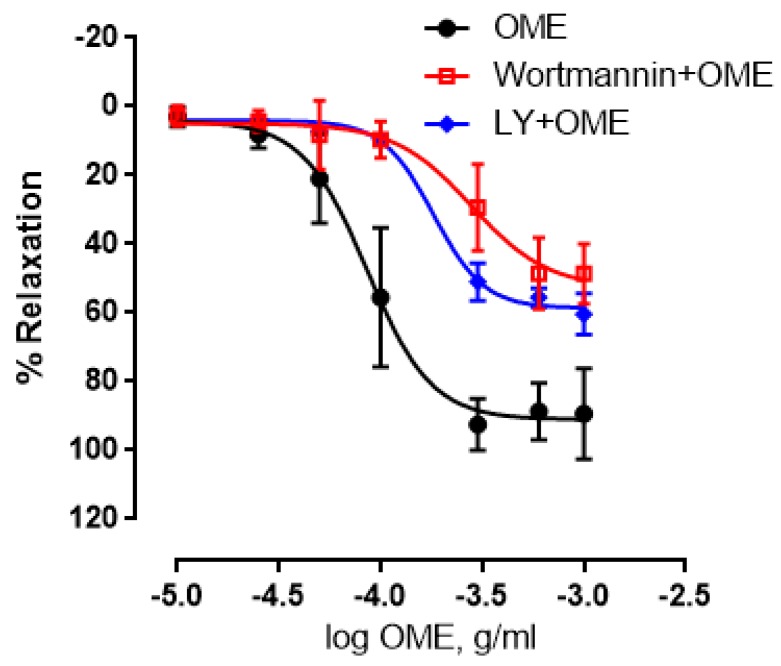
Effect of the PI3K pathway on OME-induced vasorelaxation. Rings with intact endothelium were treated with OME in the absence (black) or presence of phosphoinositide 3-kinase (PI3-K) inhibitors: Wortmannin (0.1 µM; red) or LY29400 (10 µM; blue). Data presented as mean ± SEM (*n* = 5; *p* < 0.01 for OME vs. Wortmannin + OME or LY29400 + OME).

Overwhelming evidence documents the contribution of the PI3K-Akt signaling to endothelium-dependent aortic relaxation. To determine if this signaling pathway is involved in OME-induced relaxation, we employed two inhibitors, wortmannin and LY294002. Indeed, pre-incubation with either inhibitor instigated a significant reduction in OME-induced vasorelaxation. In rings incubated in the absence of either inhibitor, the maximal relaxant effect was 91 ± 3% and pED_50_ was −4.1 with confidence interval of −4.1 to −3.9. In wortmannin pre-incubated rings, the maximal relaxant effect was 53 ± 6% (Figure 5) and pED_50_ was −3.5 with confidence interval of 3.7–3.4. Similar results were obtained when rings were pre-incubated with LY294002 (10 µM), where the maximal effect was 58%, pED_50_ was −3.7 and a 95% confidence interval of −3.8 to −6 (Figure 5). Together, our data clearly allude to the role of the PI3K signaling in OME-induced aortic relaxation.

### 3.6. Effect of K^+^ Channel Blockers on Origanum majorana Extract-Induced Relaxation

**Figure 6:**
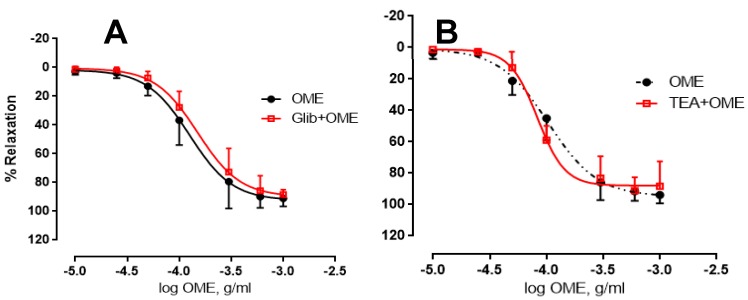
Effect of potassium channel inhibitors on OME-induced vasorelaxation. Rings with intact endothelium were treated with OME in the absence (OME; black) or presence of (**A**) 10 µM of glibenclamide (Glib + OME; red), or (**B**) 100 µM of tetraethylammonium (TEA + OME; red). Data represent mean ± SEM (*n* = 6). *p* > 0.05 for OME alone vs. either Glib + OME or OME + TEA.

Potassium channels are known to play an important role in vasodilation [24,25,26]. Thus, we wished to determine the of role of these channels in OME-induced relaxation. The vasorelaxant effects of OME were not altered by incubation with K^+^ channel blockers tetraethylammonium (TEA, 5 mM) or glibenclamide (Glib, 10 μM). Indeed, in the presence or absence of glibenclamide, the OME-induced maximal relaxant effect was 90 ± 4% or 92 ± 4% (Figure 6A), and pED_50_ was −3.7 or −4.1 with confidence intervals of −3.9 to −3.7 or −4 to −3.8, respectively. Similarly, incubation with TEA did not affect OME-induced relaxation of endothelium-intact aortic rings (Figure 6B). In the presence of TEA, the maximal effect was 88%, pED_50_ was −4, and the 95% confidence interval was −4.1 to −4. Together, these data argue for the absence of a role for potassium channels in OME-induced relaxation.

### 3.7. Effects of Ca^2+^ Channel Blockers on Origanum majorana Extract-Induced Relaxation

**Figure 7:**
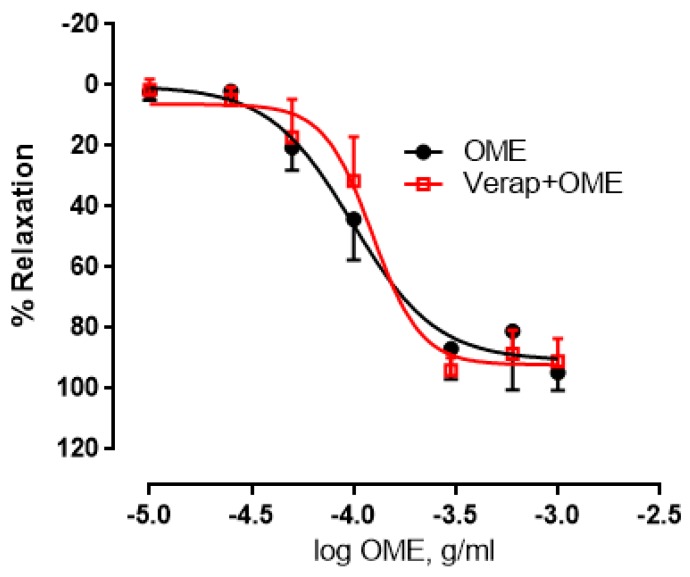
Role of calcium channels in OME-induced relaxation of aortic rings. Endothelium-intact aortic rings were pre-incubated with OME in the absence (OME; black) or presence of verapamil (verap) (1 µM; verap + OME; red). Data are presented as mean ± SEM (*n* = 5; *p >* 0.05).

To determine if calcium channels contribute to OME-induced relaxation, we pre-incubated aortic rings with verapamil (1 µM) before adding OME. There was no significant difference between the control and treated vessels (*p* > 0.05) (Figure 7). Indeed, the maximal relaxant effect in vehicle-treated or verapamil-incubated rings was not affected (91 ± 4% vs. 92 ± 3%) (Figure 7). pED_50_ was −4.0 or −3.9 with confidence intervals of −4.1 to −3.9 or −3.9 to −3.8 in the absence or presence of verapamil, respectively.

### 3.8. Effect of Atropine and Pyrilamine on Origanum majorana Extract-Induced Relaxation

**Figure 8:**
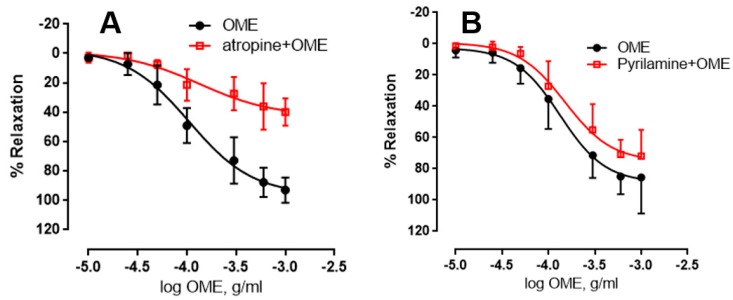
Involvement of histaminic or muscarinic receptors in OME-induced relaxation. Rings with intact endothelium were incubated with cumulative doses of OME in the absence (OME; black) or presence of (**A**) 10 µM of atropine (natural alkaloid with antagonistic properties at muscarinic acetylcholine receptors; atropine + OME; red) or (**B**) 10 µM of pyrilamine (blocker of H_1_ histamine receptors; pyrilamine + OME; red). Data showed represent mean ± SEM (*n* = 6 or 5 for atropine or pyrilamine-treated rings, respectively). *p* < 0.05 for OME alone vs. either atropine + OME; *p* > 0.05 for OME alone vs. either pyrilamine + OME.

Endothelial muscarinic M3 receptors have been shown to promote vasorelaxation by virtue of their ability to increase NO release [27]. We thus wished to examine the effect of muscarinic receptors on OME-induced relaxation. Aortic rings with intact endothelium were pre-treated with atropine (1 μM) for 30 min before NE (3 μM) pre-contraction. In the absence of atropine, the maximal relaxant effect was 96 ± 7% and a pED_50_ of −3.98 with confidence intervals of −4.1 to −3.8 (Figure 8A). However, in the presence of atropine, the maximal relaxant effect was dramatically reduced to 43 ± 9% and a pED_50_ of −3.8 with confidence intervals of −4.3 to −3.4. This clearly shows that muscarinic receptors are involved in OME-induced relaxation.

Endothelial histaminergic receptors, particularly H1 G protein-coupled receptors, are known to initiate vasorelaxation [28]. We thus investigated the influence of pyrilamine (also called mepyramine), a blocker of histamine H1-receptors on OME-induced relaxation. Compared to the control, incubation with pyrilamine did not significantly modulate OME-induced relaxation of endothelium-intact rings. In the absence of pyrilamine, the maximal relaxant effect was 88.8 ± 6.9% and a pED_50_ of −3.88 with confidence intervals of −4.0 to −3.7 (Figure 8B). In the presence of pyrilamine, there was only a marginal and insignificant reduction in the maximal relaxation value, which was noted to be 75 ± 6%. pED_50_ of pyrilamine-treated rings was found to be −3.82 with confidence intervals of −3.9 to −3.6, which is very similar to that obtained in the vehicle-treated rings (Figure 8B). Thus, histaminergic receptors do not seem to have a significant effect on OME-induced relaxation (*p* > 0.05).

### 3.9. Effect of Cyclooxygenase Pathway on *Origanum majorana* Extract-Induced Relaxation

**Figure 9:**
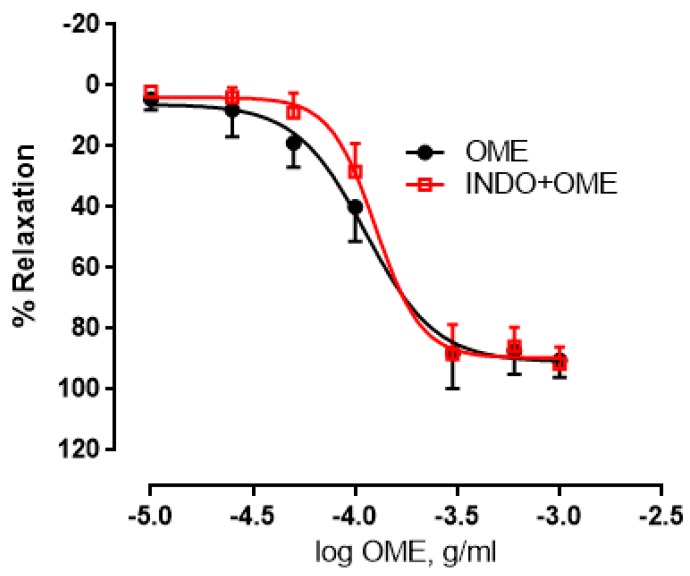
Effect of cyclooxygenase inhibition by indomethacin on OME-induced aortic relaxation. Rings with intact endothelium were pre-treated without (OME; black) or with indomethacin (a cyclooxygenase (COX)1/2 inhibitor, 10 µM; INDO + OME; red) followed by the addition of cumulative doses of OME. Data showed represent mean ± SEM (*n* = 5; *p >* 0.05).

Vascular tone is greatly affected by prostanoids generated via the activity of cyclocoxygenases [29]. We thus sought to determine the potential role of the cyclooxygenases in OME-induced relaxation. In the absence of indomethacin, the maximal relaxant effect was 90.9 ± 2.8% and a pED_50_ of −3.95 with confidence intervals of −4.0 to −3.9 (Figure 9). In the presence of indomethacin, the maximal relaxant effect was 89.6 ± 1.9% and a pED_50_ of −3.9 with confidence intervals of −3.9 to −3.8 (Figure 9). Therefore, cyclooxygenases do not play an important role in OME-induced relaxation (*p* > 0.05) (Figure 9).

## 4. Discussion

In this report, we elucidated the underlying signaling mechanisms implicated in rat aortic relaxation induced by the leaf extract of *O. majorana*, commonly known as marjoram. This plant contains a bounty of bioactives, such as thymol, carvacrol, p-cymene, sabinene hydrate, rosmarinic acid, γ-terpinene, ursolic acid, and many others [30,31]. These constituents possess antibacterial, antifungal, and antispasmodic activities, in addition to their ability to scavenge free radicals, inhibit acetylcholinesterase, and depress cardiac activity [32,33]. Some of these bioactives, like carvacrol, thymol, ursolic acid, and hesperetin indeed exert vasorelaxing activities [19,20,22,34]. In addition to these effects, we have recently shown that this plant can significantly inhibit the malignant phenotype of cancer cells [15,16]. To the best of our knowledge, this is the first report that dissects the mechanism for the vasodilator effects of marjoram. It is important to note that using the whole plant, part of it, or a crude extract may provide benefits over the use of individual bioactives. This is partly due to the notion that these herbs are commonly grown and consumed by people in rural areas where access to modern medicine is limited. In addition, these extracts can easily be prepared at home and are thus significantly less costly, making them relatively more appealing to be used. Importantly, oftentimes, the plant as a whole, or as a crude extract, may prove more effective than individual bioactives, likely due to a potential synergy between these molecules.

There is an intricate coordination between vasoconstrictors and vasodilators in regulating vasotone. The maestro of this well-orchestrated balance is the endothelial cell layer. Indeed, this layer secretes many vasoconstrictors, the most potent of which is endothelin, as well as vasodilators, the most prominent of which is NO. In endothelial cells, NO is generated from l-arginine by endothelial NO synthase (eNOS) activation, which is stimulated by the calcium-calmodulin complex or activated via the PI3K/Akt pathway [35]. The role of NO in vasorelaxation is overwhelmingly established and documented. Many stimuli, including the vascular endothelial growth factor, β-agonists, and shear-stress signals regulate NO production via a pathway involving phosphoinositide 3-kinase (PI3-K)/Akt signaling. This pathway is known to play an important role in endothelium-mediated vasorelaxation [36]. Indeed, the PI3-K/Akt pathway activates eNOS via phosphorylating serine residue at position 1177 [37,38,39]. Here, when we incubated rings with PI3-K/Akt inhibitors, there was a significant reduction in the OME-induced relaxation. In an ischemia/reperfusion injury model, it was recently reported that ginsenosides of panax ginseng promote coronary arterial flow by activating the PI3K/Akt/eNOS cascade. This establishes the cardioprotective effects conferred by isolated extracts of ginseng [40]. Similarly, in another ischemia-reperfusion injury model, stimulation of PI3K/Akt/NO cascade by a ginsenoside metabolite bestowed cardioprotection [41].

Nitric oxide is known to activate soluble guanylate cyclase (sGC) in vascular smooth muscle cells (VSMCs) and to promote the synthesis of a secondary intracellular messenger, cGMP. This cGMP elicits many biological effects including relaxation of vascular smooth musculature [42,43,44] and consequent cGMP-mediated vasodilation [45,46,47]. In this context, our data reveals that pretreatment with L-NAME, a NOS inhibitor, or ODQ, a sGC inhibitor, affect the vasorelaxant effects of OME. These results are a clear indication that the vasorelaxant effect of OME are directly related to the NO-cGMP pathway. Our results are similar to other reports showing several plant species causing aortic relaxation through NO/cGMP. Some of these plant species include *Schizophyllum commune* [48], *Salvia fruticosa* L. [7], *Euphorbia humifusa* Willd [49], *Alpiniae zerumbet* (Pers.) B. L. Burtt & R. M. Sm. (Zingiberaceae) [50], *Rhus coriaria* L. [8], and *Mansoa hirsuta* D.C. [51]. Furthermore, here in our study, we report that OME increased production of cGMP, clearly supporting the involvement of sGC-cGMP in OME-induced relaxation.

In VSMCs, the membrane potential is dynamically regulated via changes in K^+^ channel activity, which greatly influences the function of voltage-dependent calcium channels [52,53]. Interestingly, NO is known to have a direct effect on activation of the K^+^ channels [54]. Here, we employed two K^+^ channel blockers, namely glibenclamide, a highly selective blocker of ATP-sensitive K+ channels, and TEA, a blocker of voltage-sensitive K^+^ channels [55]. Our results show neither glibenclamide nor TEA appreciably modulated OME-induced effects. Thus, K^+^ channels (K^+^_ATP_ or K_V_ channels) are not involved in OME-induced aortic relaxation. This was very surprising to us due to the greatly significant role of these channels in vasorelaxation. Indeed, these channels play a critical role in regulating plasma membrane potential, and hence determining vascular tone [56,57,58]. It is already established that NO directly activates calcium-dependent potassium channels in VSMCs [59], leading to vasorelaxation by a hyperpolarization mechanism [60]. Our results argue for the absence of a role for Ca^2+^-dependent or ATP-driven potassium channels in OME-induced aortic relaxation. Our findings differ from other studies that show that certain plant extracts produce an endothelium-dependent vasodilatory effect via activation of the potassium channels. However, our results are in accordance with previous studies [61], which report that stimulation of potassium channels is not necessarily implicated in vasorelaxation. It is not unreasonable that activation of the NO/cGMP pathway produces vasodilation through alternative mechanisms.

The role of Ca^2+^ in VSMC contraction/relaxation is well-known. The two main sources for this Ca^2+^ are the extracellular space and intracellular stores like the sarcoplasmic reticulum. Other mechanisms, such as those involving protein kinase C (PKC) or Ca^2+^ sensitization, are also implicated in VSMC contractility [62]. By blocking calcium channels, danshen has been shown to potentiate endothelium-independent relaxation of rat coronary arteries [63]. However, our results show that inhibiting calcium channels did not alter OME-induced relaxation, suggesting that mechanisms independent of these channels are involved. This is similar to recently reported findings showing that L-type calcium channels may not affect extract-induced relaxation [7,8]. It is worth mentioning here that one of the limitations of this study is that we did not assess the effect of depleting extracellular calcium on the vasorelaxation involved. However, due to the fact that L-type calcium channels were not involved, one may argue that the absence or presence of extracellular calcium is unlikely to be involved.

Vasorelaxant responses initiated by histamine are known to be mediated by histamine H1 receptors [28], which are G protein-coupled receptors. In this report, we did not find any contributory role for these receptors in OME-induced relaxation. This is similar to other studies that have used other plants species such as *S. fruticosa*, *Salvia miltiorrhiza*, or *R. coriaria* [7,8,64].

Cyclooxygenases (COX-1 and COX-2) convert arachidonic acid to active prostanoids. These prostanoids are well-known to play important roles in vascular physiology and pathology, including vasorelaxatory mechanisms [65]. Prostacyclin (PGI_2_) is a potent vasorelaxant prostaglandin that is released by the endothelium. It increases the intracellular accumulation of 3′-5′-cyclic adenosine monophosphate (cAMP), which is a powerful vasodilator as well as a modulator of many mechanisms in VSMCs [66,67,68,69,70,71,72,73]. In our study, pre-treatment with indomethacin, a non-selective cyclooxygenase inhibitor, did not affect the vasorelaxant effects of OME. This result suggested that the vasorelaxant effect of OME did not have a relationship with the vascular prostacyclin pathway. This is similar to other reports showing no role for prostaglandins in relaxation of rat femoral arteries or aortic rings in response to danshen or *S. fruticosa* extract, respectively [7,74]. Similarly, we recently reported no role for these prostanoids in *R. coriaria*-induced aortic relaxation [8]. Contextually, our results are not surprising since danshen, *S. fruticosa*, *R. coriaria* and marjoram, all contain rosmarinic acid, which is known to inhibit both COX-1 and COX-2 [75].

Finally, an endothelium-independent effect may be involved, though this needs further experimentation. It is possible that the residual vasorelaxation is due to increased activity of adenylate cyclase. This pathway has indeed been reported to underlie the vasodilatory mechanism of others herbs, such as *R. coriaria* [8]. Importantly, two bioactives found in marjoram, namely kaempferol and luteolin, have been reported to contribute to vasodilatation of rat aortae by inhibiting phosphodiesterases [76]. As is well known, inhibiting phosphodiesterases leads to increased accumulation of cAMP, which itself is a potent vasodilator. However, more experiments are warranted before such an assertion is validated.

## 5. Conclusions

In conclusion, our results show that the vasorelaxant effect of OME was due to activation of the PI-3K pathway, as well as an endothelium-dependent increase in the accumulation of cGMP. Together, our findings support the use of marjoram in the management/treatment of hypertension, [17]. More research, particularly in animal models of hypertension, is needed to better support the antihypertensive potential of this herb. It is important to note that studies directed at determining which bioactives reach the bloodstream after oral consumption of marjoram or its extract are warranted.
